# 

**Published:** 1888-12-08

**Authors:** 


**Notice to Advertisers and Subscribers.**

All Advertisements, Orders for Copies of The Hospital, and Business Communications, should be addressed to The Publisher, *not to the Editor*, The Hospital, 38, Old Jewry, E.C.

**To Residents, Secretaries, and Matrons.**

The Editor will be glad to have the Regulations and each Report as issued by Asylums, Hospitals, Convalescent and Nursing Institutions, as well as those of other Charities, and an early copy in duplicate of all Appeals and Festival Notices, etc., addressed to *The Editor, The Lodge, Porchester Square, London, W.* Any superintendent, secretary, matron, or other chief official who regularly sends such information may be registered, on application to the Publisher, to receive free of cost a cover for binding The Hospital in half-yearly volumes.

### Scraps and Gleanings.

Hospital Saturday at Newbury brought in £25.

Hospital Saturday Fund will close on November 30th.

On the Mons Palatinies in Rome there stood a temple dedicated to fever.

Mrs. Jeune will again give halfpenny dinners to poor children this winter.

The Waterford Dispensary has moved into its new quarters at Peter Street.

Lord Carmarthen issues an appeal for the Skin Hospital, Leicester Square.

The Duchess of Albany has consented to become patroness of the Sanitary Institute.

The Westminster Needle Society meets twice a week at Westminster Town Hall.

The matron of Boscombe Cottage Hospital has issued an appeal for children's clothing.

There is an outbreak of diphther a amongst the servants of the Military College, Sandhurst.

The proposed amalgamation of the Irish Medical Schools causes much discussion in Dublin.

Addenbrooke's Hospital has benefited to the extent of £190 by the late bazaar at Cambridge.

A paid agent is to be appointed at Liverpool, to collect the Workmen's Hospital Saturday Fund.

A young Catholic priest named Larkin is effecting marvellous faith cures amongst the Donegal peasants.

There is to be a bazaar at Aberdeen, on December 15th, in aid of the Children's Hospital of that town

Burton-on-Trent Infirmary has benefited to the extent of £637 from the Hospital Saturday collection.

There is a serious epidemic of malignant measles at Battersea. It is proposed to close the local schools.

Barrington's Hospital, Limerick, has received £200, the receipts from the late fancy ball held in the theatre.

M. Hertenstein, President of the Swiss Confederation, has died from the effects of amputation of the right leg.

Professor Stirling is delivering the Combe lectures. His subject last week at Owens College was "The Brain."

The Frenchman Chaulfat, whose sleep of 13 days caused much excitement last year, is again in a state of trance in Soho.

Tunbridge Wells Hospital Sunday resulted in a collection of £73, given to the General Hospital, which holds 50 beds.

An entertainment was given last week by the Tradesmen's Association of Sidcup, in support of the Cottage Hospital.

Dr. Felkins' subject at the opening of the Edinburgh Health Society was "Popular Errors in Regard to Medicine."

The annual meeting of the North Staffordshire Infirmary took place on November 15th, when a satisfactory report was presented.

Seventy pounds was given to the Blackheath and Charlton Cottage Hospital, on the day of the laying of the memorial-stone.

Dartmouth Cottage Hospital has also had a ball in aid of its funds held lately, at which 147 ladies and gentlemen were present.

The death of Colonel Duncan will be a great loss to the Order of St. John of Jerusalem, of which he was for many years a member.

William Street, Chiswick, has lately been inspected, and three-fourths of the houses were found to require sanitary alterations.

Another lively meeting has been held at Londonderry about the proposed Small-pox Hospital. The cost was the subject of the quarrel.

Ray Lankester will probably obtain the Deputy-Professorship of Anatomy at Oxford, vacant by the continued ill health of Protessor Moseley.

The Hospital Saturday and Sunday of the Manchester and Salford Medical Charities resulted in a collection of £8,619, against £7,770 collected last year.

It is proposed to raise funds for the Wallingford Cottage Hospital, by inviting the children of the neighbourhood to present the institution with a Christmas-box.

Croydon General Hospital has been in luck lately: it has received five legacies during the past year, including one of £2,000 from Mrs. S. Browne, late of Sutton.

Mr. and Mrs. Drummond, of the Craiglockhart Hydropathic, Edinburgh, have entered upon a lease of the Sudbrook Hydropathic and Pension, at Petersham.

A sentence in the last report of the Birmingham General Hospital runs: "A large number of nurses have suffered from typhoid fever." Further particulars should be given.

The Grafton Street Hospital, Liverpool, has only a few patients, and fever cases are still being sent to the Workhouse Infirmary, a proceeding the Corporation ought to stop immediately.

Brighton Hospital Sunday brought in £1,630, the largest collection yet made. The County Hospital gets £888, the Dispensary £209, the Children's Hospital £226, and the West Street Institution, £137.

The Victoria Hospital for Children benefited by the Silver Fete at the Danish Exhibition to the extent of £4,000. With this sum enlargements and alterations have been made, and £1,000 invested.

The fact that Dr. Ambrose, medical officer to the dispensary of the Ardagh district, lives at Newcastle, has been brought before the Local Government Board of Dublin. All medical officers are expected to reside in their district.

At a meeting last week of the committee of the Birmingham and Midland Eye Hospital a letter was read from Mr. J. D Goodman, announcing a gift of £5,000 from an annonymous donor, £4,000 to be devoted to paying off the existing debt on the hospital, and £1,000 towards current expenditure.

### Amusements and Relaxation.

### NEW PUZZLE PRIZE SCHEME.

**COMMENCED OCTOBER 6th, ENDS DECEMBER 29th, 1888.**

On October the 6th a New Prize Scheme was commenced in these columns. Prizes will be awarded for the best Original Puzzles in Prose or Verse, relating to any of the *Social, Philanthropic, or Political Topics of the day*, and taking the form of Double Acrostics, Anagrams, Square Words, Decapitations, Transpositions, or any other Puzzles which the ingenuity of the competitors may suggest. (*Answers must accompany contributions.*)

*Readers* are requested to send in weekly the Number of Marks they consider each composition to be entitled to, three being the highest score for any one contribution.

Eight Prizes of Standard Books will be given, the choice of the book to be left to the winner.

First, Second, Third, Fourth, and Fifth Prizes for best Original Puzzles, of £1 1s., 15s., 10s. 6d., 7s. 6d., and 5s. each.

First, Second, and Third Prize for Solutions of 15s., 10s. 6d., and 5s. each.

N.B.—All contributions must reach the office, 38, Old Jewry, E.C., not later than the *First Post* on *Thursday* in each week.

### PUZZLES FOR SOLUTION.

**Eighth Week.**

No. 21. Original Double Acrostic.

We'll hope my finals always work
The reformation they profess;
So in my primals shall not lurk
A grievance without sure redress.

1. Never from the schoolboy's heart
Can love of me be banished!
From year to year I act my part,
And call to mind the vanished.

2. My voice is known to young and old
Through all the countryside;
And though I am not very bold
I'm welcomed far and wide.

3. What melancholy frame of mind
And grumpiness I bring!
To naught but gloomy thoughts inclined,
Depressed by everything!

4. You'll find me where there's work in hand;
I do it all, in fact;
'Midst students I can take my stand,
And show them how to act.

5. The genus to which I belong
Is one of many grades;
We're noted for our fragrance strong
And deep and lovely shades.

6. Geologists will know my name,
To them it should be plain;
And surely I am not to blame
If you must think again.

Patience.

No. 22. Original Anagram.—"American girl has amber."

Lady Clara.

**Marks.**

| Names. | No. of Puzzles sent in Nov. 29. | No. of Marks Nov. 29 | Total of Marks. | Names. | No. of Puzzles sent in Nov. 29. | No. of Marks Nov. 29. | Total of Marks. |
|---|---|---|---|---|---|---|---|
| Patience .... | 3 | 4 | 42 | Hercules .. | — | — | 11 |
| Lady Clara.. | 3 | 8 | 51 | Gretta ...... | 3 | 7 | 35 |
| Tinie ...... | 3 | 6 | 42 | Dandy Dick | — | — | 5 |
| Bijou ...... | 2 | 4 | 21 | Boadicea .. | — | — | 7 |
| G. D. ...... | — | — | 15 | Nipper .... | 3 | 6 | 34 |
| Thanet .... | 1 | 1 | 28 | Morico .... | 3 | 6 | 33 |
| Daisy ..... | 2 | 2 | 16 | A. M. E.... | — | — | 3 |
| Salford .... | — | — | 6 | John Gilpin | — | — | 4 |

**Answers to Sixth Week Puzzles.**

No. 16. Topical Alphabet.

| | | | | | |
|---|---|---|---|---|---|
| A. | Arab. | J. | Juggler. | S. | Season. |
| B. | Baldwin. | K. | Key. | T. | Time. |
| C. | Coin. | L. | Law. | U. | Unity. |
| D. | Dufferin. | M. | Mob. | V. | Vice. |
| E. | Erin. | N. | Noodle. | W. | Winter. |
| F. | Fawkes. | O. | Orders. | X. | Xyst. |
| G. | Good | P. | Police. | Y. | Year. |
| H. | Home. | Q. | Question. | Z. | Zeal. |
| I. | India. | R. | Rights. | | |

No. 17. Charade. In, fir, Mary = Infirmary.

**Marks for Solution of Puzzles.**

Total Marks.—Ruffles (1), 9; Daisy (1), 7; Lady Clara, 9; Reldas (1), 9; Mater (1), 5; Condorcet (1), 7; Tinie (1) 9; Gentian (2), 11; Marguerite, 5; Patience (1), 5; Gretta (1), 4; Amicus, 6; Morico, (1) 3; Scrooge (1), 10; Smut, 2; Thanet (1), 13; Jenny Wren (1), 2; Lauriston, 1.

(*For Conditions, see last week's* The Hospital.)

### A CASE FOR HELP.

"E. J. W." sends 10*s*. for this case, and "L. S." also sends 10*s*., making, up to the present time, £4 10*s*. It has now been arranged to send Bridgett to Ventnor, and it is hoped that the remaining £2 10*s*. will be sent within the next few weeks to enable him to make a sufficiently prolonged stay. Bridgett, we may mention, is not an inmate or pensioner of the Ironmongers' Almshouses, but was formerly a police-inspector on one of the railways, a position which he held for thirteen years. If he recovers sufficient strength, he will be taken on again by his former employers. He has been for two years unable to work, and the railway company has helped him and his family during the whole of that time. Donations may be sent, as before, to Mrs. Young, Matron, Ironmongers' Almshouses, Kingsland Road, E.

